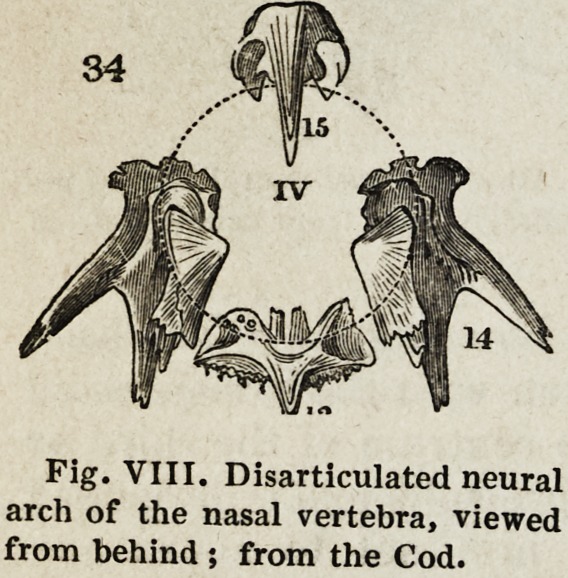# Professor Owen on the Comparative Anatomy and Physiology of the Vertebrate Animals

**Published:** 1847-04

**Authors:** 


### Art. XII.

*Lectures on the Comparative Anatomy and Physiology of the Vertebrate Animals, delivered at the Royal College of Surgeons of England, in* 1844 *and* 1846. By Richard Owen, F.R.S., Hunterian Professor and Conservator of the Museum of the College. Part I—*Fishes.* Illustrated by numerous wood-cuts.—*London,* 1846. 8vo, pp. 308.

This volume forms a portion of the promised continuation of the publication of Professor Owen's Hunterian Lectures; the first part of which, embracing the Comparative Anatomy of the Invertebrata, was noticed by us at the time of its publication in 1844. In one respect, however, this second volume has much the advantage over the first; for, whilst that was published from the notes of Mr. W. White Cooper, revised by Professor Owen, the continuation has been entirely undertaken by the Professor himself; and the result has been, that a large quantity of new matter has been added, the result of discoveries made since the delivery of the Course in 1844; whilst the views stated in that course have been substantiated and more fully developed by renewed examination of the data on which they were founded. Many details, moreover, have been added, which were necessarily omitted in the theatre, from the limited period allotted to the Lectures; so that the public are in every way great gainers by the delay, although they are yet in possession of only the first twelve Lectures of the course. The concluding volume will appear, we are led to hope, during the earlier half of the present year.

We need scarcely say that this work is one of the greatest interest and value. Of the vast opportunities at Professor Owen's command, he has most zealously availed himself; and we believe that we do but speak the general sense of competent judges when we say that, for comprehensive knowledge of the comparative anatomy of the vertebrate series, alike exact in its details and philosophical in its generalizations, he is certainly second to none, either living or dead. We should gladly take the opportunity of presenting to our readers a summary of the entire volume, in the hope of exciting their attention to its contents; but our limits compel us on the present occasion to confine ourselves to an exposition of Professor Owen's views on a question which possesses the greatest interest for the philosophical anatomist, and which should not be neglected by the anthropotomist who desires to acquire a real knowledge of the human fabric. We refer to the constitution of the vertebrate skeleton. No one can obtain the merest smattering of comparative anatomy without perceiving that mammals, birds, reptiles, and fishes, are so far constructed upon a common plan, as to agree in possessing an internal osseous skeleton, the essential part of which consists of the jointed bony casing that surrounds the spinal cord, and of the bony envelope that protects the encephalic mass. We find other parts of the skeleton deficient in the several tribes of vertebrata: thus the extremities, with the scapular and pelvic apparatus, are altogether wanting in the serpents, which moreover have no sternum; whilst the frogs and their allies are destitute of ribs, but have a sternum with well-developed anterior and posterior limbs, and scapular and pelvic arches. But we never find the vertebral column and cranial bones altogether wanting, except in those lowest fishes which retain the embryonic type so far as the development of the skeleton is concerned; and even in them we can trace the rudiments of the bony envelope to the nervous centres, in the membranous or cartilaginous tube which incloses the cerebro-spinal axis. But as we pursue our studies further, we find that although a cranium and vertebral column are thus constantly present, they exhibit considerable variations amongst different animals, not merely in regard to their form, but as to the number of parts of which they are made up. We find, for example, that the cranium of a cod has a vastly greater number of separate bones than that of man; and that the single vertebra of the human back-bone is represented in the shark by a number of pieces held together by ligaments only. The comparative anatomist, who would duly systematize his study, will not be satisfied with bringing together all the diverse forms which he encounters in his progress through the vertebrated sub-kingdom; but he will endeavour, by patient scrutiny and careful comparison, to form an *ideal type* of a vertebra, which shall include all the parts that seem essential to its structure, and of which he may consider the several forms that present themselves to his attention as *variations,* resulting from want of development, from excessive development, or even (in some instances) from multiplication of certain of the elements.

This search after the "typical vertebra" has been made by several distinguished comparative anatomists, especially Carus and Geoffroy St. Hilaire; but the result of Professor Owen's inquiries is not precisely accordant with the conclusions of either of these distinguished physiologists, and seems to us much more satisfactory. There is a vagueness about Carus's conception of a vertebra, which must be apparent to any one who attempts to follow out his interpretation of the bones of the extremities, in which he uses the term "vertebra" in a sense almost equivalent to that of the more general term "bone" or "segment;" whilst, on the other hand, Geoffroy St. Hilaire has founded his idea of the vertebrate structure too exclusively upon that form of it which is presented in the fish, and has consequently included in it some elements which it should not really embrace, whilst he has neglected others which form legitimate parts of the vertebra in other classes.

Before proceeding, however, with the analysis of the vertebrate skeleton, we shall inquire, with Professor Owen, into its relations with the tegumentary envelope or dermo-skeleton, which is the usual characteristic of the invertebrata. The latter, of which we have the most remarkable example in the shell of the crustacean, is formed by the consolidation of the epidermis, and serves for the protection of the entire body; like the epidermis it is non-vascular and is not capable of interstitial growth; so that if it be not formed (as is the case with the shell of the echinus) of separate plates capable of being extended at their edges, or if it be not so shaped that additions made to its free borders give increased dimensions to the interior cavity (as is the case with the shells of mollusca, which form only a partial envelope to the body), it must be periodically thrown off and renewed, in order that it may be adapted to the augmentation of the body during the period of growth. Accordingly we find the "moulting" process almost universal in the articulated series, whose bodies and limbs are enveloped in a tightly-fitting integument. The contrast between the skeletons of the vertebrated and invertebrated animals is most striking, when their respective relations to the nervous system are considered. In none of the latter does the nervous apparatus attain that predominance which it possesses in the former; we find its centres isolated from each other, and without any constant or determinate position. Thus the number and the relative situation of the ganglia varies in the mollusca with the development of the foot and of different portions of the muscular layer of the mantle, and with the position of the branchiæ; all of which conditions are subject to such variation, that scarcely any two genera are alike in the arrangement of their nervous centres. And in insects, crustacea, and other articulata, although the ganglia are usually disposed on a more uniform plan, their number varies with that of the segments of the body, and their relative size with that of the development of the parts of the muscular apparatus with which they are respectively connected. We nowhere encounter anything like the same fixity of plan as is shown in the disposition of the cerebro-spinal axis of vertebrata, from the very lowest to the highest of the group; nor does the entire mass of the nervous centres present in any case the same bulk relatively to that of the body, as it possesses in all vertebrata, excepting perhaps the very lowest of the series. In the invertebrata, accordingly, we find that the nervous system only receives the same amount of support and protection from the skeleton as the other tissues possess; for although it has been thought that certain internal projections of the dermo-skeleton of the insect and crustacean were specially adapted for this purpose, yet there is really no conformity between these and the number of ganglia, and they are so situated as to inclose the intercommunicating cords rather than the ganglia themselves; so that, as Professor Owen very justly urges, they must be regarded as essentially destined for the attachment of muscles; their relation to the nerve-trunks being accidental. On the other hand, the internal skeleton of the vertebrata, as we have seen, is essentially connected with the nervous system; the support and protection of the nervous centres being obviously its primary purpose; and the number of pieces of which the vertebral column (which constitutes its fundamental portion) is composed, being in constant relation with the number of pairs of nerves to be given off from the nervous axis. There is a similar relation between the functions of the nervous system, and those of the endo- and exo-skeletons respectively. When the powers of discerning hurtful agencies by the organs of sense, and of avoiding them by the use of the motor apparatus, are dull and contracted, the entire animal is protected by a hard insensible dermal armour; but as those powers become expanded and quickened, the body is disencumbered of its coat of mail, the skeleton is put inside and made subservient to muscular activity, and the skin becomes proportionally more susceptible of outward impressions of pleasure and pain. Thus, as Professor Owen justly remarks, "the exo-skeleton is the reflex of the circumambient medium and relations of the animal; the endo-skeleton is the index of its motive energies and its intelligence."

We need not waste our space in exposing the unphilosophical nature of the views of those, who have attempted to identify as "homologous" parts a segment of the thoracic dermo-skeleton of an articulated animal, and a thoracic segment of a vertebrated animal, including (with the vertebra) a pair of ribs and a segment of the sternum. For although this identification has been advanced by authorities no less weighty than Geoffroy St. Hilaire and Carus, and although it may seem to derive support from a cursory comparison of the two structures, yet it totally fails, when the examination is pushed further, and is found to depend upon a relation of "analogy" merely, which results from an adaptation of each to a set of functions in some degree similar.*

The most convincing proof of the want of real conformity between the endo- and exo-skeletons, is their coexistence in no inconsiderable number of vertebrated animals. Thus among fishes, we find the lepidosteus and the ostracion entirely covered with a connected armour of dense enamelled bony scales; the substance of which, though formed by the calcification of the cutaneous tissues, presents the same organized structure as the bones of the internal skeleton. In the crocodiles, many of the dermal plates present almost the same complete ossification; and the tesselated armour of the armadillos and extinct glyptodons affords even in the mammalian class an example of the coexistence of a well-developed bony envelope, with a complete osseous internal skeleton. And among the highest invertebrata, we find somewhat of the same coexistence; the true homologue of the endo-skeleton presenting itself in the cuttle-fish as a cartilage supporting the cephalic ganglia; whilst the exo-skeleton of the testaceous mollusks is represented by the calcareous dorsal plate.

But there are certain hard parts, both in vertebrated and invertebrated animals, which cannot be referred to either of these divisions,—to the endo- or neuro-skeleton, or to the exo- or dermo-skeleton. Thus we find in the lobster, a calcified framework supporting the gastric teeth and giving attachment to the muscles that work them; and in the bulla there is a pair of calcareous plates imbedded in the walls of the muscular stomach or gizzard, which add considerably to their crushing and triturating power. A corresponding group of parts is to be found in the general skeleton of most vertebrated animals.

"The cartilages or bones of the larynx, trachea, and bronchi of air-breathing vertebrates, the bones and cartilages supporting the branchiæ in fishes and batrachians, the bones in the hearts of certain birds and mammals, are examples of the visceral series of hard parts, or the "splanchno-skeleton" as it has been termed by Carus; and very nearly and naturally connected with this primary division of hard and dry parts are those bones and gristles which form capsules or support the appendages of the special organs of the senses; as for example, the sclerotic osseous cups or plates of the eye, the petrous capsule of the labyrinth,

* We follow Professor Owen in his employment of the terms *homology* and *analogy*, as expressive of relations essentially distinct. Homologous organs are those which correspond with each other in position, connexions, and development, although they may differ in function; thus, the lungs of air-breathing vertebrata and the swimming-bladder of fishes are indubitably homologous, although the latter has a function almost invariably distinct from that of the former. This relation has been before expressed by the terms "essential" or "fundamental analogy;" but there is a great advantage in the use of the simple term "homology." On the other hand, "analogous" organs are those which have similar functions in different animals, or in different parts of the same animal, although they may be formed of elements really dissimilar. Thus, the wings of bats, birds, flying-fish, and pterodactyles are analogous organs, and are so far homologous as being supported by some modification of the bones of the anterior extremity; but though analogous, they are not homologous with the wings of the Draco volans, which are expanded over an extension of the false ribs; and still less are they homologous with the wings of insects, which are supported by prolongations of the dermo-skeleton.

the ossicles and cartilages of the tympanum and external ear, the turbinate bones and gristles of the nose. But some of these "sense-capsules" are connected and intercalated with the true bones of the endo-skeleton, and subservient to similar functions, besides their own special uses; so that they are generally described as ordinary bones of the skull. As in all arrangements of natural objects, where nature is followed in selecting their characters, so in classifying the parts of the general skeleton of vertebrata, the primary groups blend into one another at their extremes, and make it difficult to draw a well-defined boundary line between them. Thus, the hyoids and branchial arches closely resemble one another in fishes. Bones of the dermo-skeleton combine with those of the endo-skeleton to form the opercular and the single median fins. But we must not on that account abandon the advantage of arrangement and classification, in acquiring an intelligible and tenable knowledge of a complex system of organs, when typical characters clearly indicate the general primary groups. Clearly appreciating the existence of such characters in the very numerous and diversified parts of the general skeleton of the vertebrate animals, I therefore adopt the primary division of those parts into endo-skeleton, exo-skeleton, and splanchno-skeleton." (p. 24.)

It must be carefully borne in mind that the presence of *bone* is not requisite to indicate the existence of a constituent part of the skeleton. There are many instances in which parts that are ossified in some animals are cartilaginous or merely fibrous in others. Thus every intelligent anatomist is aware that the costal cartilages of the mammalia are ossified in birds, so as to form sternal ribs; and that white lines across the recti abdominis represent the abdominal ribs of reptiles. In the lower cartilaginous fishes, such as the lamprey and myxine, no part of the endo-skeleton advances beyond the cartilaginous grade; and in the amphioxus or lancelet it is entirely fibrous. In regard to the composition of bone, we may remark, that the recent analyses of Von Bibra and Dr. Stark indicate that the proportions of animal and mineral matter do not vary so widely in the bones of different animals, or in those of young and old individuals, as was formerly supposed; the difference in texture being principally due to the quantity of membranous and adipose matter in the canals and cancelli of bone (forming no part, however, of the true osseous texture) and to the variable proportion of water. It is on this last condition that the relative softness of the bones of the sharks and rays depends; for although they are commonly ranked as cartilaginous fishes, they are not so in reality; their bones, whilst soft and flexible, being but little different in composition from the densest parts of the skeleton of higher animals, except in losing a much larger proportion of their weight by drying, and in containing a greater proportion of the soluble salts of soda.

After describing the general nature, chemical constitution, development, growth, and structure of the osseous system, Professor Owen next proceeds to define *a bone;* the endeavour to do which, he remarks, has not been the least difficult part of his task, with reference to the applicability of the definition to the vertebrate series in general.

"To the human anatomist the question—what is *a bone?*—may appear a very simple, if not a needless one; he will most probably reply that *a bone is any single piece of osseous matter entering into the composition of the adult skeleton;* and, agreeably with this definition, he will enumerate about 260 bones in the human skeleton. Soemmering, who includes the thirty-two teeth in his enumeration, reckons from 259 to 264 bones; but he counts the os spheno-occipitale as a single bone, and also regards, with previous anthropotomists, the os temporis, the os sacrum, and the os innominatum as individual bones; the sternum, he says, may include two or three bones, &c.: but, in birds, the os occipitale is not only anchylosed to the sphenoid, but these early coalesce with the parietals and frontals; and, in short, the entire cranium consists, according to the above definition, of a single bone. Blumenbach, however, applying the human standard, describes it as composed of the proper bones of the cranium consolidated, as it were, into a single piece. And in the same spirit most modern anthropotomists, influenced by the comparatively late period at which the sphenoid becomes anchylosed to the occipital in man, regard them as two essentially distinct bones. In directing our survey downwards in the mammalian scale, we speedily meet with examples of persistent divisions of bones which are single in man. Thus it is rare to find the basi-occipital confluent with the basi-sphenoid in mammalian quadrupeds; and before we quit that class, we meet with adults in some of the marsupial or monotrematous species, for example, in which the supra-occipital, 'pars occipitalis propriè sic dicta' of Soemmering, is distinct from the condyloid parts, and these from the basilar or cuneiform process of the os occipitis; in short, the single occipital bone in man is four bones in the opossum or echidna; and just as the human cranial bones lose their individuality in the bird, so do those of the marsupial lose their individuality in the ordinary mammalian and human skull. In many mammalia we find the pterygoid processes of anthropotomy permanently distinct bones; even in birds, where the progress of ossific confluence is so general and rapid, the pterygoids and tympanics, which are subordinate processes in man, are always independent bones. In many mammalia, the styloid, the auditory, the petrous, and the mastoid processes remain distinct from the squamous or main part of the temporal throughout life; and some of these claim the more to be regarded as distinct bones, since they obviously belong to different natural groups of bones in the skeleton; as the styloid processes, for example, to the series of bones forming the hyoidean arch. The artificial character of that view of the os sacrum, in which this obviously more or less confluent congeries of modified vertebræ is counted as a single component bone of the skeleton, is sufficiently obvious. The os innominatum is represented throughout life in most reptiles by three distinct bones, answering to the iliac, ischial, and pubic portions in anthropotomy. The sternum in most quadrupeds consists of one more bone than the number of pairs of ribs which join it; thus it includes as many as thirteen distinct bones in the *Bradypus didactylus*." (pp. 36-7.)

From these and numerous similar facts, we are led to see the arbitrary character of any definition of a bone founded upon the composition of the skeleton in any single animal, the complex nature of many of the so-called single bones of man, and the real independence of many of those parts which are only ranked as "processes" in anthropotomy. We further see that it is only by a comparative examination of *all* the forms presented by the vertebrate skeleton, that we can learn what are to be regarded as really individual bones, and what modifications they undergo in the human subject. That a fundamental unity prevails throughout, is obvious not only from the gradation which may be traced between forms apparently the most diverse, but also from the fact that the similarity among all becomes much greater when the comparison is instituted at an early period of development. Thus in the human fœtus the expanded portion of the occipital bone is ossified from four distinct centres, which correspond to the four permanently distinct bones of the marsupials and reptiles; the pterygoid processes have distinct centres of ossification; the styloid and mastoid processes, and the tympanic ring, are separate parts in the fœtus; the constituent vertebræ of the sacrum remain distinct to a later period; and the ilium, ischium, and pubis are still later in anchylosing to form the os innominatum. So strongly impressed was Cuvier with the importance of attention to the osteogenic process, in determining the true composition of the skeleton, that he goes so far as to assert that, in order to ascertain the true number of bones in each species, we must descend to the primitive osseous centres as they are manifested in the fœtus. According to this rule, we ought to count the humerus as three bones, and the femur as four bones, instead of one; for the ossification of the latter begins at four distinct points,—one for the shaft, one for the head, one for the great trochanter, and one for the distal condyles. But there is no such distinction in any of the lower classes; for in both birds and reptiles the femur is developed from a single centre. The rule laid down by Cuvier would greatly mislead us therefore, if rigidly applied; and it is necessary to distinguish, as Professor Owen justly points out, between those centres of ossification which have *homological* relations, and those that have only *teleological* ones: i. e. between the separate points of ossification of a human bone which typify permanently distinct bones in the lower animals, and the separate points which, without such signification, facilitate ossification, and have for their final cause the well-being of the growing animal. The contrast between these relations is well seen in the following examples.

"The young lamb or foal can stand upon its four legs as soon as it is born; it lifts its body well above the ground, and quickly begins to run and bound. The shock to the limbs themselves is broken and diminished at this tender age by the divisions of the supporting long bones,—by the interposition of the cushions of cartilage between the diaphyses and the epiphyses. And the jar that might affect the pulpy and largely-developed brain of the immature animal, is further diffused and intercepted by the epiphysial articular extremities of the bodies of the vertebræ. We thus readily discern a final purpose in the distinct centres of ossification of the vertebral bodies, long bones, and the limbs of mammals, which would not apply to the condition of the crawling reptiles. The diminutive brain in these low and slow cold-blooded animals does not demand such protection against concussion; neither does the mode of locomotion in the quadruped reptiles render such concussion likely; their limbs sprawl outwards, and push along the body, which commonly trails upon the ground; therefore we find no epiphyses with interposed cartilage at the ends of a distinct shaft in the long bones of saurians and tortoises. But when the reptile moves by leaps, then the principle of ossifying the long bone by distinct centres again prevails, and the extremities of the humeri and femora long remain epiphyses in the frog.

"A final purpose is no doubt also subserved in most of the separate centres of ossification which relate homologically to permanently distinct bones in the general vertebrate series; it has long been recognized in relation to facilitating birth in the human fœtus; but some facts will occur to the human osteogenist, of which no teleological explanation can be given. One sees not, for example, why the process of the scapula which gives attachment to the pectoralis minor, the coraco-brachialis, and the short head of the biceps, should not be developed by continuous ossification from the body of the blade-bone, like that which forms the spinous process of the same bone. It is a well-known fact, however, that not only in man, but in all mammalia, the coracoid process is ossified from a separate centre. In the monotremes it is not only distinct, but is as large a bone as in birds and reptiles, in which it continues a distinct bone throughout life. Here, then, we have the homological, without a teleological explanation of the separate centre for the coracoid process in the ossification of the human blade-bone." (pp. 38-9.)

The distinction here first pointed out by Professor Owen is of primary importance, and must be kept in view in all our attempts at a philosophical determination of the essential parts of the vertebrate skeleton; for without such a guide we shall be continually thrown aground—as past philosophical anatomists have been—in the comparison of the bones of animals in which the skeleton seems most complex (owing to the great number of pieces of which it is composed), with those of animals in which (owing to the much smaller amount of distinct pieces) it appears to be most simple. There are certain bones which are *simple,* as being developed from a single centre through the entire series, and the determination of the homologies of these presents no difficulty; but the representatives of the *compound* bones, or of those which are developed from two or more separate centres, cannot be always found in groups of simple bones. For if these bones are only *teleologically compound,*—that is, if their development from separate centres has reference only to the special exigencies of a particular animal or class, as is the case with the cylindrical bones in the mammalia,—we shall find them elsewhere represented by simple bones. Indeed we may even find a group of bones remaining permanently distinct, as that which composes each half of the lower jaw in fishes and icthyoid reptiles, and yet represented elsewhere by a simple bone; the teleological purpose or final cause having required this subdivision in a particular tribe, whilst no traces of it are to be found elsewhere. On the other hand, the *homologically compound* bones are those which, like the occiput, scapula, vertebræ, and sacrum, are developed from separate centres, which represent permanently distinct simple bones in other vertebrata; and thus their relations extend over the whole vertebrate series. The comparison on which we shall presently enter, between the vertebrate skeleton of fishes and that of man, will afford us examples of both these species of relation.

In order to understand the fundamental type of the vertebrate skeleton, we must commence its study,—not in that form of it in which there is the greatest heterogeneousness of character, on account of the variety of purposes to which it has to be rendered subservient,—but in the class in which vegetative uniformity most prevails, and the primitive type is least obscured by teleological adaptations. Such conditions are best displayed in the skeletons of fishes; in which, although they are apparently the most complex (from the number of distinct pieces of which they are usually composed), we find a greater real simplicity, since these pieces are for the most part the *simple* bones, by the union of which, under various forms, the *homologically compound* bones of higher animals are made up. Moreover, it is much more easy to detect in this class the intercalation of osseous pieces from the dermo- and splanchno-skeletons among the parts of the neuro-skeleton; all traces of which are frequently lost, if we confine our attention solely to the skeletons of air-breathing vertebrata. But, as Professor Owen justly observes, fishes form only one branch of the vertebrate stem, which, like other primary branches, ramifies in diverging from the common trunk; and we should miss our aim, and be led astray from the detection of the true general type of the vertebrate skeleton, were we to confine our observations to fishes alone, which have teleological adaptations and other peculiarities of their own.

"A comparison of their skeletons with those of the higher classes teaches that the natural arrangement of the parts of the endo-skeleton in vertebrata, like that of the exo-skeleton in articulata, is in a series of segments succeeding each other in the axis of the body. I do not find these successive segments composed of precisely the same number of bones in all vertebrata; rarely, indeed, in the same animal. Yet certain constituent parts of each segment do preserve such a constancy in their existence, relative position, and offices throughout the body, as to enforce a conviction that they are homologous parts, both in the consecutive series of the same individual skeleton, and throughout the entire series of vertebrate animals. To each of these primary segments of the skeleton I shall, with Geoffroy St. Hilaire, apply the term 'vertebra:' the word may seem to the anthropotomist to be used in a different or more extended sense than it is usually understood; yet he is himself, unconsciously, perhaps, in the habit of including in certain vertebræ of the human body elements which he excludes from the idea in other natural segments of the same kind; influenced by differences of proportion and coalescence, which are the most variable characters of a bone. Thus the rib of a cervical vertebræ is the 'processus transversus perforatus,' or the 'radix anticus processus transversi vertebræ colli;' whilst in the chest, it is 'costa' or 'pars ossea costæ.' But the ulna is not less an ulna in the horse, because it is small and anchylosed to the radius." (pp. 41-2.)

Fig. I. Segment of endo-skeleton, *mammal.*

A vertebra, then, according to Professor Owen's definition, is *one of those segments of the endo-skeleton which constitute the axis of the body, and the protecting canals of the nervous and vascular trunks;* such a segment may also support *diverging appendages.* Now as all hope of detecting the true homologies of the vertebræ in the assemblage of bones which form the cranium, and of identifying the really homologous parts in animals that are formed upon plans greatly dissimilar,—still more, of ascertaining the fundamental character of the extremities, depends upon a true determination of the real elements of a vertebra, this point is one of essential importance. The following is Professor Owen's account of the composition of a complete typical vertebra, excluding the diverging appendages:

*c.* A body or *centrum.*

*n.* Two *neurapophyses.*

*p.* Two *parapophyses.*

*pl.* Two *pleurapophyses.*

*h.* Two *hæmapophyses.*

*ns.* A *neural spine.*

*hs.* A *hæmal spine.*

Fig. II. Ideal typical vertebræ.

"These, being usually developed from distinct and independent centres, I have termed 'autogenous' elements. Other parts, more properly called processes, which shoot out as continuations from some of the preceding elements, are termed 'exogenous;' e. g. (*t*) the diapophyses or upper 'transverse processes,' and (*z*) the zygapophyses, or the 'oblique' or 'articular processes' of human anatomy.

"The autogenous processes generally circumscribe holes about the centrum, which, in the chain of vertebræ, form canals. The most constant and extensive canal is that (Fig. 2, *n*) formed above the centrum, for the lodgment of the trunk of the nervous system (neural axis) by the parts thence termed 'neurapophyses.' The second canal (Fig. 2, *h*) below the centrum, is in its entire extent more irregular and interrupted; it lodges the central organ and large trunks of the vascular system (hæmal axis), and is usually formed by the laminæ, thence termed 'hæmapophyses.' At the sides of the centrum, most commonly in the cervical region, a canal (Fig. 3, *v*) is circumscribed by the pleurapophysis or costal process (Fig. 3, *pl*), and by the diapophysis or upper or transverse process (Fig. 3, *t*), which canal includes a vessel, and often also a nerve." (p. 43.)

The mode in which these elements are arranged in the thoracic vertebræ of mammalia, will be seen by reverting to the first figure, in which are marked the neural spine or spinous process (*ns*), the neurapophyses or neural arches (*n*), the zygapophyses, or oblique processes (*z*), the diapophyses or upper transverse processes (*d*), and the centrum or body (*c*), of which the mammalian thoracic *vertebra* is commonly regarded as consisting; and in addition we have the parapophyses or anterior roots of the the hæmapophyses (*h*) represented by the *costal cartilage;* and the hæmal spine (*hs*) by the *sternum,* which, though usually flat in the mammalia, often presents a projecting keel as in birds. The great enlargement of the hæmal arch is here due to the increased development of the pleurapophyses; transverse processes (*p*), and the pleurapophyses (*pl*), making up the *ribs;* and by the removal of the hæmapophyses from the centrum, and their articulation with the distant ends of the pleurapophyses. Besides the neural and hæmal canals, above and below the centrum, we find the lateral vascular canals, included between the origin of the rib and the included between the transverse process, which in the cervical region are two roots of the transverse process itself, of which the inferior one is obviously the pleurapophysis here anchylosed to the body of the vertebra.—In the cervical vertebra of a bird, we find another arrangement of these elements; the hæmapophyses being anchylosed to the under part of the centrum, and the hæmal canal being only required for the protection of the carotid arteries. In the cranium, we shall find the chief modification to result from the expansion of the neural arch to form the brain case, just as the hæmal arch in the thoracic region is expanded to include the heart and lungs in the thoracic region.

Fig. III. Natural typical vertebra from the neck of a Pelican.

We shall not follow Professor Owen through his description of the vertebral column in fishes; but shall only observe that he clearly demonstrates the fallacy into which Geoffroy St. Hilaire was led, when he adopted the vertebra of the fish as the type of vertebral structure in general; the hæmapophyses being always absent or unossified, the parapophyses being frequently so modified as to inclose the hæmal canal; whilst, on the other hand, certain bones are superadded, which have an apparent claim to be ranked among the elements of the vertebræ, but which really belong to the dermal skeleton. Thus we find the dorsal fins of osseous fishes supported by a row of bones superposed upon the ordinary neural spines, and imagined by Geoffroy St. Hilaire to be formed by the change in the position of the two lateral halves, which were in his view placed one on the top of the other, instead of meeting on the median line. But it is now generally admitted that these spines in reality belong to the dermo-skeleton, although they are closely related in position and aspect to the real neural spines of the vertebræ.* But there are other projections from the vertebræ of certain fish, which constitute "diverging appendages" of the vertebra itself; such are certain spines, which are to be met with in the salmon and herring, the mackerel tribe and the dolphin, projecting from the ribs near their heads and sometimes diverging from the parapophyses and even from the neurapophyses. The homologue of these diverging appendages may be distinctly recognized in the ribs of birds; each of which, as is well known, is furnished with a process that passes over the ribs next below, and serves to give additional firmness and compactness to the bony case. We shall presently find that the recognition of them in the cranial vertebræ of fishes conducts us, according to Professor Owen, to the true homology of the bones of the extremities.

The fact that the spines which support the dorsal fin, constituting a second row of greater or less extent above the true neural spines, belong to the dermo-skeleton, is extremely well seen in the sturgeons, which have a well-developed osseous endo-skeleton coexisting with a covering of hard enamelled calcareous plates; and to this tribe the philosophical anatomist finds it requisite to make frequent reference, for the determination of the parts that really belong to each division. Here we find the rays upon which the dorsal fin is supported, clearly developed from the dermal plates, which along the middle line of the back shoot upwards and backwards a moderately long spine. From the base of these dermal spines, other spines usually shoot downwards into the intervals of the neural spines; these inverted interneural spines, which are double in the flat-fish, appear to be regarded by Professor Owen as formed by the "vegetative repetition" of the neural spines themselves; but we must take leave to question this determination, for it seems to us much more natural to consider them as portions of the dermo-skeleton passing inwards,—the manner in which they are intercalated among the true neural spines bearing a very strong resemblance to the reception of the fangs of the teeth into the alveolar processes of the jaw. It is not only in the back that we find these additional parts derived from the dermo-skeleton; for just as in the framework of the dorsal fin we find interneural spines and dermoneural spines, so in that of the anal fin we recognize interhæmal spines and dermohæmal spines. The framework of the caudal fin is composed of similar intercalary and dermal spines, superadded to the proper neural and hæmal spines of the proper caudal vertebræ, which have coalesced and been shortened by absorption, in the progress of embryonic development, to form the base of the terminal fin. There is usually an exact correspondence in intimate structure, between these dermal spines and the real bones which support them; this conformity has been urged by Professor Agassiz as an argument against the possibility of drawing a valid distinction in such cases between the parts belonging to the neuro-skeleton and those which appertain to the dermal envelope. But such a conformity exists likewise between the undoubted tegumentary scales of the lepidosteus

* In the herring, moreover, we find a sort of sternum formed by dermal plates, which are articulated to the ends of the ribs.

and its true internal bones; so that the presence of true bony structure must not be regarded as of itself proving that the part which exhibits it belongs to the neuro-skeleton; this being only determinable by its connexions and by the history of its development.

We shall now follow Professor Owen, as briefly as may be consistent with clearness, through his view of the craniology of the osseous fishes; in which group, owing to the permanent distinctness of a large proportion of the elements of which the skull is composed, the vertebral structure may be recognized more clearly than in any other.

"The bones of the skull are primarily divided in anthropotomy into those of the cranium and those of the face; but the proportions which these divisions bear to one another in man are reversed in fishes. According to this binary classification, the facial series in fishes includes an extensive system of bones—the hyoid—of which part only, viz., the 'styloid element,' is admitted into the skull by the anthropotomist, who describes it as a process of the 'temporal bone.' This very 'temporal,' moreover, is originally and essentially an assemblage of bones, which are always distinct in fishes and reptiles; and the squamous part, which enters so largely into the formation of the cranial cavity in man and most mammals, has no share in its formation in the lower vertebrata. The two classes of cranial and facial bones having been originally founded upon the exclusive study of the most peculiarly and extremely modified skull in the whole vertebrate series—that of man,—their characters, as might be expected, are artificial, and applicable to the same bones in only a small proportion of the vertebrata; the unity of the plan pervading the organization of which, it is the business of the anatomist, properly so called, to demonstrate.

"The bones of the skull of fishes are primarily divisible into those of

A. Neuro-skeleton;
B. Splanchno-skeleton;
C. Dermo-skeleton.

"The bones of the neuro- or proper endo-skeleton are arranged here, as in the rest of the body, in a series of horizontally succeeding segments; each segment consisting of an upper (neural) and a lower (hæmal) arch, with a common centre, and with diverging appendages. As the bones respectively entering into the formation of these segments are the same in relative position, and nearly the same in number, as in the typical vertebræ of the trunk—the excess arising from subdivision of the peripheral elements—the same term ought to be extended to those cranial segments which has been usually restricted to their neural arches, in which the typical characters of the vertebra are least departed from." (pp. 86-8.)

Before proceeding with the enumeration of the parts entering into the composition of the cranial vertebræ, we must stop to notice that Professor Owen fixes the number of these vertebræ (which has been a subject of much discussion) at four; and that he justifies this by reference to the encephalon, which consists of four primary divisions, succeeding each other horizontally in a linear series. These are,—1, the medulla oblongata, with the superimposed cerebellum, recently termed by Vogt the *epencephalon;* 2, the third ventricle, with its upper (pineal) and lower (hypophysial) prolongations, and the superimposed optic lobes, or the *mesencephalon;* 3, the cerebrum proper, or *prosencephalon;* and 4, the olfactory ganglionic prolongation of the cerebrum, or *rhinencephalon.* Though subject to various degrees of anchylosis, the cranial vertebræ of fishes always agree in number with these primary divisions of the encephalon, and are named by Professor Owen in accordance with them.

Fig. IV. Disarticulated bones of the cranial vertebræ, and sense-capsules, in Cod-fish; the hæmal arches (H, H) and appendages in diagrammatic outline.

"Each cranial vertebra, or natural segment of the skull, is divided into a *neural arch*, with which the centrum and parapophyses are always more immediately connected; and a *hæmal arch*, with its appendages.

"The neural arches are:

I. Epencephalic arch (Figs. IV and V, 1, 2, 3, 4);
II. Mesencephalic arch (Figs. IV and VI, 5, 6, 7, 8);
III. Prosencephalic arch (Figs. IV and VII, 9-12);
IV. Rhinencephalic arch (Figs. IV and VIII, 13-15).

"The hæmal arches are:

i. Scapular, or scapulo-coracoid (Fig. IV, in outline, 50-52);
ii. Hyoid or stylo-hyoid (Ditto, 25, 38-43);
iii. Mandibular or tympano-mandibular (Ditto, 25-32);
iv. Maxillary, or palato-maxillary (Ditto, 20-22).

"The appendages of the hæmal arches are:

1. The pectoral (Fig. IV, in outline, 54-57);
2. The branchiostegal (Ditto, 44);
3. The opercular (Ditto, 34-37);
4. The pterygoid (Ditto, 23-24).

"The bones of the *splanchno-skeleton* constitute:

The ear-capsule or petrosal, and otolite (Fig. IV, 16, 16');
The eye-capsule or sclerotic, and pedicle (Ditto, 17);
The nose-capsule or æthmoid, and turbinal (Ditto, 18-19);
The branchial arches (Ditto, 45-49).

"The bones of the *dermo-skeleton* are:

Supra-temporals (Fig. IV, 71);
Supra-orbitals (Ditto, 72);
Sub-orbitals (Ditto, 73, 73');
Labials (Ditto, 74)." (pp. 88-9.)

We shall now examine each of these divisions separately. In most osseous fishes, the bones encompassing, or in vertebral relation with, the epencephalon, and thus forming the neural arch of the first or occipital vertebra, are six in number, as shown in Fig. V. These are called, in

Fig. V. Disarticulated epencephalic arch, viewed from behind, in the Cod.

ichthyology, the basi-occipital (1), the ex-occipitals (2, 2), the supra-occipital (3), and the par-occipitals (4, 4). No one can have any difficulty in recognizing in the basi-occipital the centrum or body of a vertebra, since it articulates posteriorly with the body of the atlas; and bearing in mind the relations of these bones to the nervous cord, it is obvious that the ex-occipitals are the neurapophyses, the par-occipitals the parapophyses, and the supra-occipital (which very frequently possesses a prominent keel or ridge superiorly) the neural spine. Among the variations in form presented by these bones in different members of the class, are some which indicate in a very striking manner their conformity to the vertebral elements of the spinal column. On the other hand, various degrees of anchylosis are met with, which, by uniting two or more pieces, apparently reduce the number of parts of the vertebra; and in the polypterus the elements of the neural arch are all united into one piece, which corresponds to the occipital bone of man. In the early condition of the latter, we find seven ossific centres; one for the basilar portion or body of the vertebra, two for the condyles or neurapophyses, two below the crucial ridge for the parapophyses, and two above for the triangular portion which forms the summit of the bone and represents the neural spine. The supra-occipital, its homologue in fishes, is itself divided by a median suture in the lepidosteus, showing that it is in like manner formed from two centres of ossification.

The neural arch of the second cranial vertebra surrounding the mesencephalon, is composed of seven bones, of which the parietal elements in the human cranium are the largest, hence this vertebra is termed the parietal vertebra. Its body or centrum is formed by the *basi-sphenoid* (Fig. VI, 5); its neurapophyses are the bones termed the *ali-sphenoids* (6); its parapophyses are the *mastoid* bones (8); whilst its spine is formed by the *parietals* (7), which in the fish are comparatively small, in accordance with the small size of the encephalon, whose upper portion they are to protect. The names given to these bones sufficiently indicate the parts of the human cranium with which they are homologous. The basi-sphenoid is always united by continuous ossification with the *pre-sphenoid* (9), which is considered by Professor Owen as the centrum of the third or frontal vertebra; and the fact that the whole basi-pre-sphenoid is developed from a single centre of ossification is shown by him not to afford any sufficient objection to this homology, since many other cases exist in which bones that are elsewhere undoubtedly distinct, are in like manner repre-

Fig. VI. Disarticulated neural arch of parietal vertebra, viewed from behind; from the Cod.

sented by a single bone developed from one ossifying centre. The elements of the occipital and parietal vertebræ are so formed as to leave a large cavity, or *otocrane*, for the lodgment of the proper acoustic capsule; this cavity, which is analogous to the orbital cavity for the lodgment of the eye, is excavated in the ex-occipital, par-occipital, ali-sphenoid, and mastoid bones, with the addition in some instances of the parietal and supra-occipital. The acoustic capsule is either cartilaginous or osseous; when in the latter state it is known as the *petrosal* bone; and although it coalesces with the elements of the neuro-skeleton in higher animals to form the temporal bone, yet we think that Professor Owen is perfectly justified in regarding it as in itself a portion of the splanchno-skeleton, like the sclerotic capsule of the eye.

The neural arch of the third or frontal vertebra, which surrounds the prosencephalon of fishes, has for its centrum (as already stated) the pre-sphenoid (Fig. VII, 9); its neurapophyses are the *orbito-sphenoids* (10), whose essential functions are the protection of the sides of the prosencephalon, and the transmission of the optic nerve; the *post-frontals* (12) form its parapophyses; whilst the *frontal* (11), which is oftener divided by a median suture than a single bone, obviously constitntes its spine. We thus see the exceedingly complex nature of the human sphenoid bone; since, independently of the pteregoid processes, whose representatives have not yet come before us, the upper portion entering into the walls of the cranial cavity is represented in the fish by the basi-pre-sphenoid, the ali-sphenoids, and the orbito-sphenoids, which enter into the composition of two distinct vertebræ. All these are anchylosed into one bone in the polypterus.

Fig. VII. Disarticulated neural arch of frontal vertebra, viewed from behind; from the Cod.

The circle of bones which completes the axis of the skull anteriorly, and protects the olfactory ganglia, may be regarded as the neural arch of the fourth or nasal vertebra. Its body, however strange this may appear to the mere anthropotomist, formed by the *vomer* (Fig. VIII, 13); which, instead of being a narrow plate that occupies scarcely any space on either side of the median plane, is here a broad thick bone, whose aspect presents no difficulty in its recognition as the centrum of a vertebra. The neurapophyses are formed by the *prefrontals* (14), which defend and support the olfactory prolongations of the cerebral axis, and bound the orbits anteriorly; and the spine is formed by the *nasal* bone (15), which is usually

Fig. VIII. Disarticulated neural arch of the nasal vertebra, viewed from behind; from the Cod.

single. The parapophyses are not present as distinct elements in this vertebra. The elements of this nasal vertebra are closely connected with the capsule of the organ of smell, which is represented in man by the æthmoidal and turbinal bones. The former is seldom completely ossified even in the osseous fishes; and it often retains its original cartilaginous condition. By Oken and Bojanus, who regarded the cranium as made up of only three vertebræ, the æthmoid was regarded as the centrum or body of the third or anterior vertebra. This view might appear justifiable, when we look merely to the form and position of this bone in the higher vertebrata; but a more comprehensive examination shows that it forms the anterior wall rather than the floor of the cranium; and that it is related rather to the protection of the olfactory organ, than to the support of the olfactory ganglia, although these sometimes rest upon it as in man. The existence of other sense-capsules as parts of the splanchno-skeleton, affords an obvious reason for regarding the boundary of the olfactory organ in the same light.

We have thus endeavoured to explain the principal features of Professor Owen's determination of the homologies of the neural arches of the cranial vertebræ; and we feel a very strong conviction that it is the most philosophical that has yet been offered. It is founded upon the relation of the cranial envelopes to the nervous centres and nerves proceeding from them; and the slightest acquaintance with the anatomy of the encephalon in fishes enables us to see that the number of its principal segments is four. It is very interesting to perceive that this number corresponds with that of the parts entering into the composition of the cephalic ganglia in the myriapoda; and that the number of Professor Owen's cranial vertebræ is identical with the number of segments detected by Mr. Newport in the cephalic portion of the head of those animals. (See Brit. and For. Med. Rev., Vol. XX, p. 493.) The determination of the complex system of bones forming the remainder of the skull of the fish, and including those which are subservient to the respiratory process, is much more difficult; and it must be entirely guided by that of the bodies and neural arches of the cranial vertebræ. Our space will only admit of our indicating very briefly the results of Professor Owen's inquiries on this point.

The *hæmal* arches, like the neural, are four in number; and are connected with the lower portions of the neural arches. The most anterior appertaining to the nasal vertebra, is the *palato-maxillary* arch; of which the *palatines* (Fig. IV, 20) constitute the pleurapophyses, the *maxillary* (21) the hæmapophyses, and the *intermaxillary* or *premaxillary* (22) the hæmal spine. This arch has a "diverging appendage" consisting of the *pteregoid* (24) and *ento-pteregoid* bones (23); which are, however, by no means constantly present. The ten bones of which the palato-maxillary arch is made up in osseous fishes are very commonly so disposed as to appear like three parallel and independent arches, successively attached behind one another by their keystones to the forepart of the axis of the skull, and with their piers or crura suspended freely downwards and outwards. But this arrangement seems to have reference to the peculiar mobility which the lips of fish, in the absence of other prehensile organs, require to possess; and the existence of only one true arch in this series of palato-maxillary bones seems to be indicated by its simple condition in the lepidosiren and in plagiostomous fishes, as well as by the fact that it is completed or closed inferiorly at one point only, viz., where the premaxillaries meet and coalesce.

The *tympano-mandibular* arch presents its true inverted or hæmal character; its apex or keystone being formed by the symphysial junction of the lower jaw hanging downwards freely below the vertebral axis of the skull. It is usually, however, very complex in its construction; each of the vertebral elements being here repeated or subdivided, so as to be made up by many distinct bones. Thus the pleurapophyses are represented by the *epi-tympanics* (Fig. IV, 25), the *meso-tympanics* (26), the *pre-tympanics* (27), and the *hypo-tympanics* (28); but even within the class of fishes we find a tendency to simplification by the coalescence of the four bones on each side into two; and there seems no difficulty in regarding this complicated tympanic pedicle of the lower jaw as a "serial repetition" of the same element which, as the pedicle of the upper jaw, forms the single palatine bone of either side. The *mandible* or lower jaw consists of two principal portions on each side; of which the one that is articulated to the suspensory pedicle (29) represents the hæmapophysis; whilst the anterior portion (32) in which the teeth are implanted evidently corresponds with the hæmal spine. The hæmapophyses often possess, however, one or two additional pieces, which are found still more developed in reptiles. The extreme mobility given to the lower jaw by this subdivision of the arch, is one of the most remarkable *teleological* modifications in the cranium of fishes; and if viewed in this light, we are spared the necessity of looking out for a separate *homology* for each element, which would certainly not be found in animals that have no occasion for such a peculiar conformation. One of the most interesting, and in our opinion the most successful, of Professor Owen's determinations, is that of the homology of the *opercular* bones which support the gill-cover. This series, made up of the *pre-opercular* (Fig. IV, 34), the *opercular* (35), the *sub-opercular* (36), and the *inter-opercular* (37), is now regarded by him as the "diverging appendage" of the tympano-mandibular arch. Physiologists have long since repudiated the strange doctrine propounded by Geoffroy St. Hilaire of the identity of these large bones with the minute *ossicula auditûs* of higher animals; but their connexion with the proper elements of the cranium has always been doubtful; and the idea suggested long since by Professor Owen himself,—that these bones really belong to the dermo-skeleton—has been accepted by many distinguished anatomists. He has found, however, upon further inquiry, that this is not their true relation; and looking at the size and importance which these diverging appendages from the hæmal arch elsewhere possess in the vertebral column of the fish, we have little doubt that his present view is the correct one.

The third inverted arch of the skull, the *hyoidean*, is the hæmal arch of the parietal vertebra; being suspended (through the medium of the epi-tympanics) from the mastoid bones or parapophyses. Like the preceding arch, it is usually composed of portions more numerous than the ordinary vertebral elements which they represent; the pleurapophyses being represented by the *stylo-hyals* (Fig. IV, 38), the hæmapophyses by the *epi-hyals* (39) and the *cerato-hyals* (40), whilst the hæmal spine is made up of the *basi-hyals* (41), the *glosso-hyals* (42), and the *uro-hyals* (43). That these pieces only form a single arch, however, is perfectly obvious from their relative position; and we find in some fishes a considerable simplification of it. The "diverging appendage" of the hyoidean arch retains the form of a series of simple, elongated, slender, slightly-curved rays, which are articulated to depressions in the outer and posterior margins of the hæmapophyses. They are called *branchiostegals,* or gill-cover rays, because they support the membrane which closes the branchial chamber externally. The number and size of these rays vary considerably; sometimes they are absent altogether, whilst in the elops there are thirty on each side, and in the angler they are of enormous length. The most common number is seven, as in the cod (Fig. IV, 44). With the keystone or hæmal spine of the hyoidean arch are connected, more or less closely, a series of bony arches, of which six are usually at first developed and five retained. They are altogether called the *branchial* arches; but only the first four support the gills; the fifth, which is beset with teeth and guards the opening of the gullet, is distinguished as the pharyngeal arch. All these gill-and-tooth-bearing arches appertain to the splanchno-skeleton, or to that category of bones to which the hard jaw-like pieces supporting the teeth of the stomach of the lobster belong. They are sometimes cartilaginous when the true endo-skeleton is ossified, as in the lepidosiren; they are never ossified in the perenni-branchiate batrachia, and are the first to disappear in the larvæ of the caduci-branchiate species; and both their place and mode of attachment to the skull demonstrate that they have no essential homological relation to its vertebrate structure.

The fourth hæmal arch, appertaining to the occipital vertebra, is the *scapular;* forming the framework to which the anterior or thoracic extremities are attached. However absurd such a notion may appear to the mere anthropotomist, it is fully justified by an examination of the condition of this arch in the class of fishes; for, except in the higher cartilaginous fishes, it is as distinctly articulated to the cranium at its hinder part, as the lower jaw is in front. The pleurapophysis is usually represented by two bones; the *supra-scapula* (Fig. IV, 50) and the *scapula* (51); these are always confluent in the siluroids. The hæmapophysis is formed by a bone which is commonly termed the clavicle, but which Professor Owen regards (and we think with good reason) as rather corresponding with the *coracoid* of other oviparous vertebrata. The hæmal spine is wanting; the lower end of the arch being completed by the symphysis of the carocoids, which are usually united by a ligament; but are sometimes joined by a dentated suture. Like the other hæmal arches of the cranial vertebræ, the scapular arch supports a "diverging appendage" on each side; and this diverging appendage is nothing else than the *thoracic extremity.* We can well anticipate the ridicule with which this determination will be received by those who delight in laughing at what they are pleased to call the outrageous absurdities of the philosophical anatomists,—merely because they cannot comprehend them. That the hands and arms of man are nothing else than "diverging appendages" to his occipital bone, will be doubtless in their eyes to stamp the whole system as a tissue of dreamy transcendentalism. But if such persons will go to Nature, and interrogate her by a careful and candid scrutiny of the various forms and combinations which she presents, with the real desire to ascertain whether there be a guiding plan, a unity of design, throughout the whole, or whether each organism is built up for itself alone without reference to the rest,—we are confident that they will find the former doctrine to be irresistibly forced upon them; and if, having adopted it, they will further inquire into the particular mode in which this plan is worked out, and will follow the guidance of the distinguished Hunterian Professor in the examination of the cranial bones of fishes, we are quite certain that if they do not feel every probability of his general correctness, they will at least be unable to prove him to be in error on any important point. We speak this advisedly, after having been present at a long debate between Professor Owen and the greatest ichthyologist of the present or any other time, Professor Agassiz; in which we perceived that every objection which the latter could urge against the vertebral theory (to which he *had been*, though we doubt whether he *still can be*, a decided opponent) had been met by anticipation in Professor Owen's system, and that he was consequently able to afford a satisfactory solution of it.

The homology of the thoracic extremities as "diverging appendages" of the scapular arch, is manifestly free from objection on the score of their complexity of structure, when we trace them to their simplest form, such as is presented in the lepidosiren, in which there is but a single-jointed ray. We have but to suppose these rays to be multiplied laterally,—according to the law of vegetative repetition, which most affects the parts that are farthest removed from the centre,—in order to understand how the bone which is single in the arm becomes double in the fore-arm, and forms a quintuple or even more numerous series in the hand. And when we look at the size and number occasionally presented by the branchiostegal rays, and the expanded fin-like aspect and movement of the opercular bones, we can scarcely deny that whatever be the relation which *they* bear to the hæmal arches of the two anterior cranial vertebræ, that of the thoracic extremities is the same to the scapular arch, or hæmal arch of the occipital vertebra. Nor does the removal of the scapular arch and its appendages from the back of the skull to the other end of the neck, in the higher vertebrata, afford the least ground for imagining that their homology is thereby changed; for this removal is seen to take place even in the class of fishes; and in the embryonic condition of the higher vertebrata, the scapular arch closely approximates the occiput, almost as in ordinary fishes. Numerous other examples of such displacement might be cited; it will be sufficient to refer to the ventral fins of fishes, which are not less the homologues of the pelvic extremities of the higher vertebrata, because they are sometimes brought forwards into close proximity with the pectoral fins, or even into advance of them.

Besides the cranial vertebræ with their various appendages, and the auditory, ophthalmic, and nasal sense-capsules, the skull even of the osseous fishes contains some bones that are referred by Professor Owen to the dermo-skeleton. The evidence for this homology is chiefly derived from the cranium of the sturgeon, in which the dermo- is much more fully developed than the neuro-skeleton; and also from the fact that these bones are more especially connected with the mucous organs of the skin. They are those denominated the *sub-orbital*, the *supra-orbital*, and the *supra-temporal*. One of the sub-orbitals is folded upon itself in such a manner as to form a mucous channel, which extends from the orbit to the nasal sac, and is obviously analogous to the lachrymal canal of higher vertebrata; hence it may be inferred that the lachrymal bone, which has the same position and connexions, has the same origin, being the only part of the dermo-skeleton which is ossified in man, unless the turbinate bones are to be regarded in the same light.

We should most gladly quote largely from Professor Owen's admirable remarks on the teleology of the skeleton of fishes, or the modifications it presents in conformity with the special conditions in which these animals are to exist. The whole plan of structure appears at first sight to be so different from that which prevails in higher vertebrata, that if we do not keep the necessity for these modifications steadily in view, we shall be continually baffled in our homological pursuit. We must content ourselves, however, with one extract.

"We must guard ourselves from inferring absolute superiority of structure from apparent complexity. The lower jaw of fishes might at first view seem more complex than that of man, because it consists of a greater number of pieces, each ramus being composed of two or three, and sometimes more, separate bones. But, by parity of reasoning, the dental system of that jaw might be regarded as more complex, because it supports often three times or ten times, perhaps fifty times the number of teeth which are found in the human jaw. We here perceive, however, only an illustration of the law of vegetative repetition as the character of inferior organisms; and we may view in the same light the multiplication of pieces of which the supporting pedicle of the jaw is composed in fishes. But the great size and double glenoid or trochlear articulation of that pedicle, are developments beyond and in advance of the condition of the bones supporting the lower jaw in mammalia, and relate both to the increase of the capacity of the mouth in fishes for the lodgment of the great hyoid and branchial apparatus, and to the support of the opercula or doors which open and close the branchial chambers. The division of the long tympanic pedicle of osseous fishes into several partly-overlapping pieces adds to its strength, and by permitting a slight elastic bending of the whole diminishes the liability to fracture. The enormous size, moreover, of the tympano-mandibular arch, and of its diverging appendages, contributes to ensure that proportion of the head to the trunk which is best adapted for the progressive motion of the fish through the water. But, without the admission and appreciation of these pre-ordained adaptations to special exigencies in the skeleton of fishes, the superior strength and complex development of the tympanic pedicle and its appendages would be inexplicable and unintelligible in this lowest and firstborn class of vertebrate animals." (pp. 151-2.)

We have only left ourselves space to add, that the other organs and systems are treated in the same comprehensive manner with the osseous, although not with the same fullness of detail,—the determination of the homologies of the cranial bones being a question of such deep interest to the palæontologist as well as to the philosophical anatomist, as to require a full investigation of the data on which it is founded. We may especially remark that it gives us very great satisfaction to find that Professor Owen's views on the composition of the nervous centres of fishes are, in all essential particulars, the same with those which have been recently expressed in our own pages; and that his deductions regarding the physiology of the cerebellum, from the comparative development of that organ in different groups, are the very same with those at which we had arrived.

We anticipate with great interest the remaining volume, which is to include the comparative anatomy of reptiles, birds, and fishes; and we trust that Professor Owen may not be driven, by the numerous demands upon his time and attention, to that system of indefinite postponement, which has manifested itself of late in the delay of the later portions of almost every anatomical work that has been published in detached parts or volumes. Having ventured upon something like a promise as to the time of its appearance, we hope that he will not indulge the belief that literary "promises, like pie-crust, are made to be broken."

---

## Figures and Tables

**Fig. I. f1:**
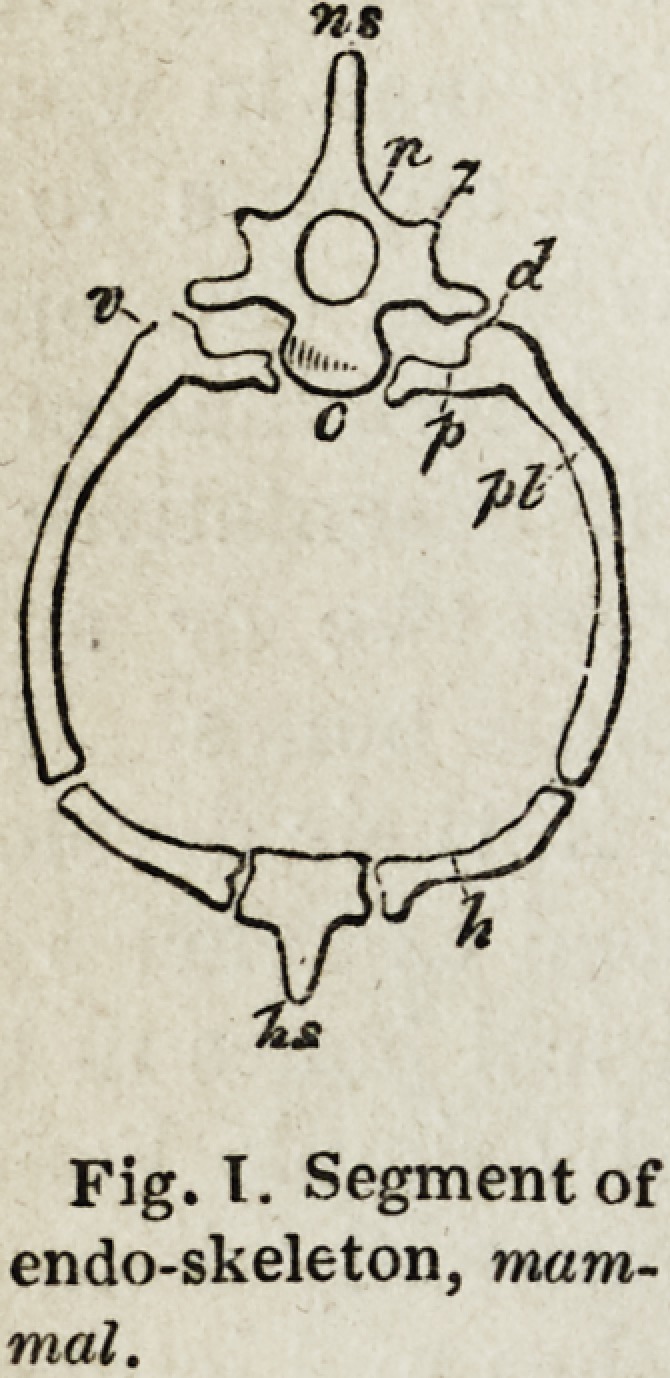


**Fig. II. f2:**
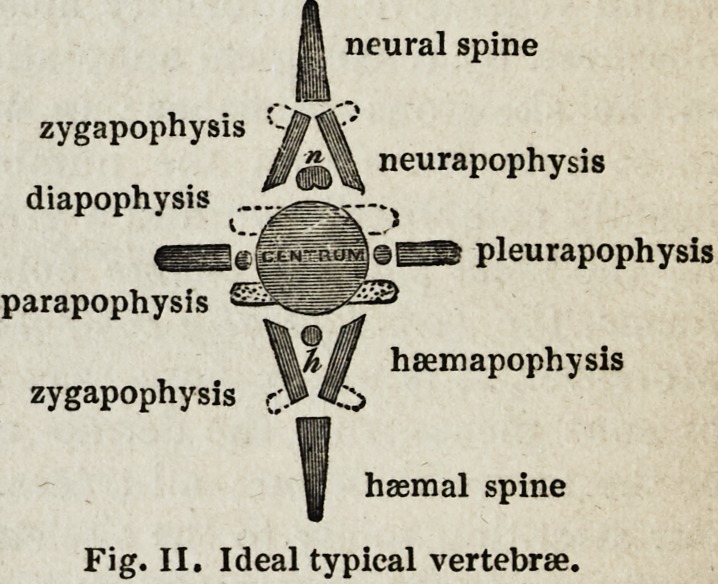


**Fig. III. f3:**
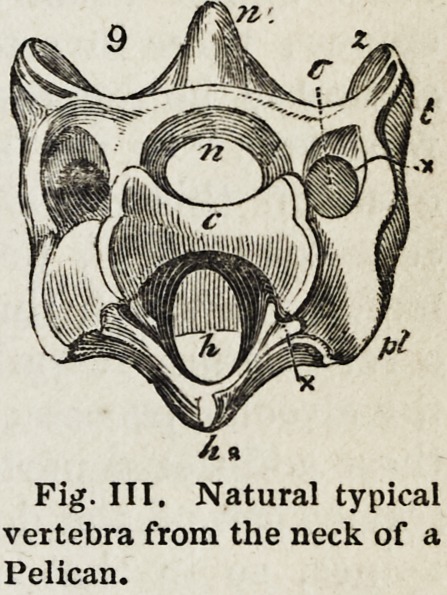


**Fig. IV. f4:**
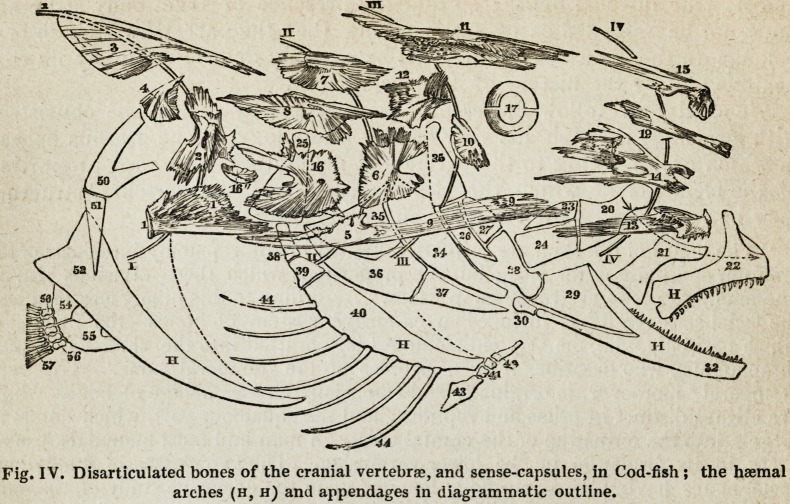


**Fig. V. f5:**
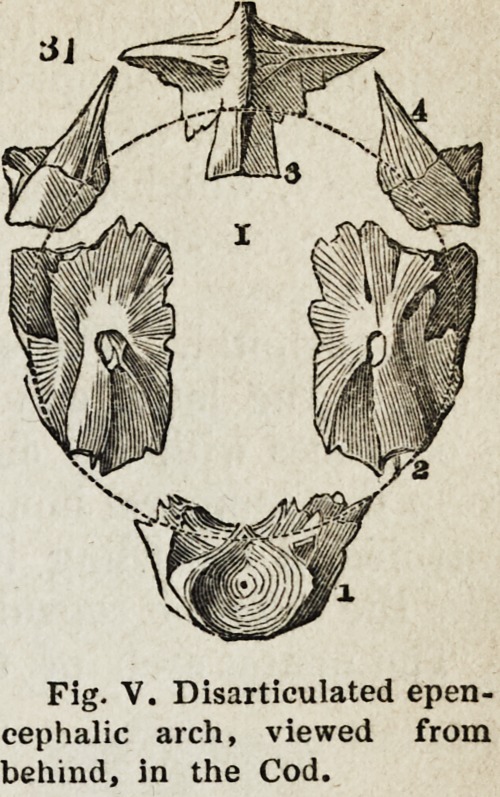


**Fig. VI. f6:**
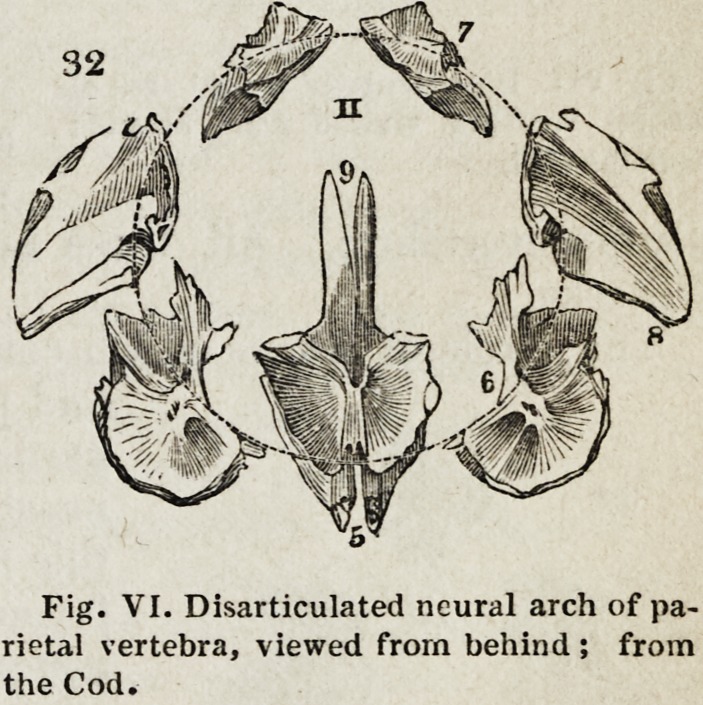


**Fig. VII. f7:**
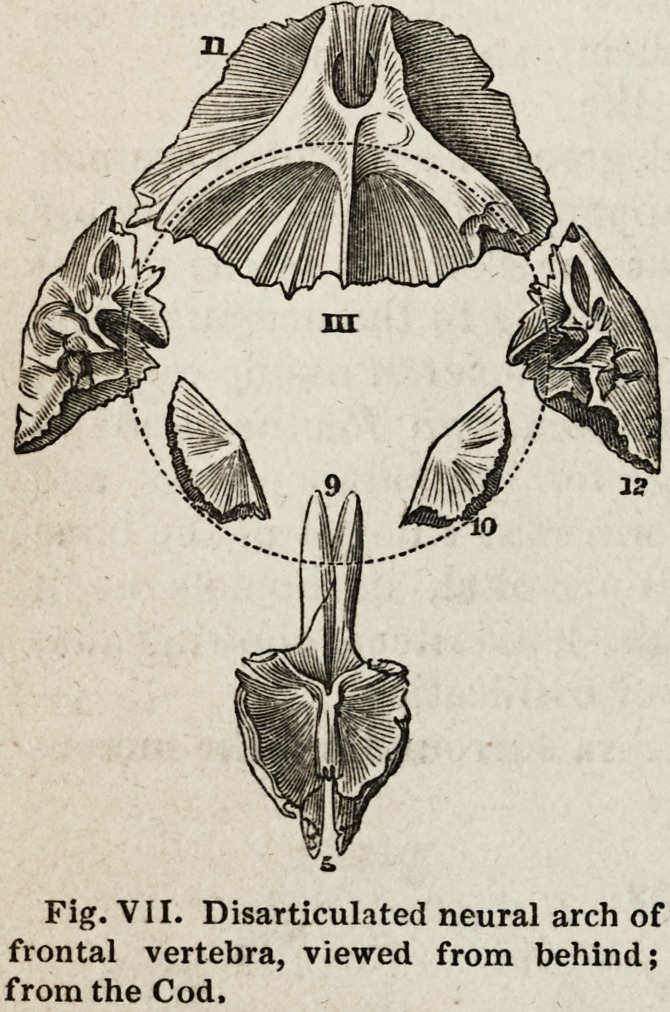


**Fig. VIII. f8:**